# Founder *BRCA1*/*BRCA2*/*PALB2* pathogenic variants in French-Canadian breast cancer cases and controls

**DOI:** 10.1038/s41598-020-63100-w

**Published:** 2020-04-16

**Authors:** Supriya Behl, Nancy Hamel, Manon de Ladurantaye, Stéphanie Lepage, Réjean Lapointe, Anne-Marie Mes-Masson, William D. Foulkes

**Affiliations:** 1 Department of Human Genetics, McGill University, Montreal, Quebec Canada; 2 Research Institute of the McGill University Health Centre, Montreal, Quebec Canada; 3 Centre de recherche du Centre hospitalier de l’Université de Montréal and Institut du cancer de Montréal, Montreal, Quebec Canada; 4 Department of Medicine, Université de Montréal, Montreal, Canada; 5 Institut du cancer de Montréal, Montréal, Québec Canada; 6 Department of Medical Genetics, Jewish General Hospital, Montreal, Quebec Canada

**Keywords:** Genetics, Population genetics, Breast cancer

## Abstract

Inherited germline pathogenic variants are responsible for ~5% of breast cancer globally. Through rapid expansion and isolation since immigration in the early 17^th^ century, French Canadians are a relatively genetically homogenous founder population and therefore represent a unique demographic for genetic contributions to disease. To date, twenty variants in *BRCA1*, *BRCA2*, and *PALB2* that predispose families to breast and ovarian cancer have been identified as recurring in the French-Canadian founder population. Our objective was to evaluate the clinical efficacy and validity of targeted genetic testing for these variants in Montreal French Canadians. A total of 555 breast cancer cases unselected for family history or age of diagnosis were genotyped, along with 1940 controls without a personal or family history of cancer. A Sequenom genotyping assay identified a pathogenic variant in 0.2% (5 of 1940) of cancer-free controls, and 3.8% (21/555) of breast cancer cases. Almost 10% (12/113) of early onset cases were heterozygous for founder *BRCA1* or *BRCA2* pathogenic variant. Of twenty variants tested, only seven were identified in this study. The option of providing this test as population-based screening is discussed.

## Introduction

Breast cancer is the most prevalent cancer worldwide (excluding non-melanoma skin cancer) and the second leading cause of death by cancer in women^1^. A strong family history of breast and/or ovarian cancer, as well as a personal history of early-onset or bilateral breast cancer, are suggestive of a heterozygous germline variant in a high-risk breast cancer susceptibility gene. *BRCA1* and *BRCA2* are the canonical genes in this family^2^, and the risks for breast cancer to age 80 among female *BRCA1* or *BRCA2* heterozygotes is 72% and 69%, respectively^3^. Partner and localizer of *BRCA2* (*PALB2*) is essential to homologous recombination repair in response to double-stranded breaks in DNA^4^ and is a more recently identified breast cancer predisposing gene. Average cumulative risk of developing breast cancer with a pathogenic germline *PALB2* mutation is approximately 35% by age 70, and risk is higher in women with a family history of breast cancer (58%)^5^.

French Canadians descended from French settlers to Canada in the 17^th^ century. Since then, settlers from countries other than France have immigrated to Canada, but early French founders contribute more to the present genetic landscape^6^. Variants more prevalent in the French-Canadian population have been identified in established breast cancer predisposition genes *BRCA1*, *BRCA2*, and *PALB2*^7–14^. For many years, genetic testing for recurrent pathogenic variants (Table 1) was routinely offered to Montreal French Canadian women with or at high risk for breast cancer via McGill University cancer genetics clinics. This approach was recently replaced with more broad-based panel testing in keeping with global trends.

**Table 1:** Twenty likely pathogenic variants in *BRCA1*, *BRCA2*, and *PALB2* in French Canadians that predispose to breast and ovarian cancer.

Gene	Reference Transcript	Historical nomenclature	HGVS Nomenclature	Predicted Protein Change	Source
*BRCA1*	NM_007294.3	1081 G > A	c.962 G > A	p.(Trp321Ter)	Oros *et al*. (2004)
		1135insA	c.1016dupA	p.(Val340GlyfsTer6)	Rudkin *et al*. (2006)
		2080insA	c.1961dupA	p.(Tyr655ValfsTer18)	Simard *et al*. (2007)
		2244insA	c.2125_2126insA	p.(Phe709TyrfsTer3)	Simard *et al*. (2007)
		2953del3insC	c.2834_2836delGTAinsC	p.(Ser945ThrfsTer6)	Tonin *et al*. (1998)
		3768insA	c.3649_3650insA	p.(Ser1217TyrfsTer2)	Tonin *et al*. (1998)
		3875del4	c.3756_3759delGTCT	p.(Ser1253ArgfsTer10)	Oros *et al*. (2004)
		4446 C > T	c.4327 C > T	p.(Arg1443Ter)	Tonin *et al*. (1998)
		5221delTG	c.5102_5103delTG	p.(Leu1701GlnfsTer14)	Tonin *et al*. (2001)
		E352X	c.1054 G > T	p.(Glu352Ter)	Simard *et al*. (2007)
		Q1846X	c.5536 C > T	p.(Gln1846Ter)	Cavallone *et al*. (2010)
*BRCA2*	NM_000059.3	2816insA	c.2588dupA	p.(Asn863LysfsTer18)	Tonin *et al*. (1998)
		3034del4	c.2806_2809delAAAC	p.(Ala938ProfsTer21)	Oros *et al*. (2004)
		3398del5	c.3170_3174delAGAAA	p.(Lys1057ThrfsTer8)	Oros *et al*. (2006)
		3773delTT	c.3545_3546delTT	p.(Phe1182Ter)	Oros *et al*. (2004)
		6085 G > T	c.5857 G > T	p.(Glu1953Ter)	Tonin *et al*. (1998)
		6503delTT	c.6275_6276delTT	p.(Leu2092ProfsTer7)	Tonin *et al*. (1998)
		8765delAG	c.8537_8538delAG	p.(Glu2846GlyfsTer22)	Tonin *et al*. (1998)
		E3002K	c.9004 G > A	p.(Glu3002Lys)	Cote *et al*. (2012)
*PALB2*	NM_024675	Q775X	c.2323 C > T	p.(Gln775Ter)	Foulkes *et al*. (2007)

Full gene panel screening of all known breast cancer susceptibility genes is an effective way to identify gene variants in populations where a large number of different and rare mutations exist, many often unique to a single family. However, in founder populations such as French Canadians, a *de novo* mutation or a mutation present in a founding member can become widespread in later generations, contributing significantly to the overall disease burden. Genetic testing focused on founder variants can be a cost-effective and sensitive assay for high-risk French-Canadian breast cancer patients in Quebec^7^.

Founder variants were previously screened in a series of 192 Montreal French Canadian breast cancer cases unselected for age of diagnosis or family history of cancer. Only three of seven variants tested were identified in the series^15^. Additional subsets of these variants were also genotyped in highly selected French-Canadian breast cancer cases^9,10^. Therefore, there has not been a comprehensive genotyping study for all twenty variants in a large series of Montreal breast cancer cases.

Here we present the population frequency of 20 variants in Montreal French Canadian invasive breast cancer cases unselected for family history or clinicopathological features, as well as in a cohort of French-Canadian cancer-free controls. Understanding the contribution of specific founder variants to breast cancer risk in such a population will help to assess the feasibility of conducting population-wide genetic testing for variants that are over-represented in this population.

## Results

A total of 26 heterozygotes were identified: five heterozygotes among controls (5/1940, 0.26%), and 21 in cases (21/555, 3.78%). Of the five heterozygotes in controls, two carry a pathogenic variant in *BRCA1*, and three carry different pathogenic variants in *BRCA2*. All heterozygotes identified in cancer-free controls were men. In breast cancer cases, four *BRCA1* heterozygotes and 17 *BRCA2* heterozygotes were identified. There were no heterozygotes of the *PALB2* c.2323 C > T variant in our cases or controls. There were no cases or controls that were heterozygotes for more than one variant. Three variants were significantly associated with breast cancer: *BRCA2* c.3170_3174delAGAAA (p.Lys1057ThrfsTer8), c.3545_3546delTT (p.Phe1182Ter), and c.8537_8538delAG (p.Glu2846GlyfsTer22). Thirteen variants were not observed in any samples tested (Table 2).

**Table 2:** Germline heterozygous variants identified in cases and controls.

Gene	HGVS Nomenclature	Controls			Cases	*p*-value*
		Males	Females	All		
*BRCA1*	c.962 G > A	0/879	0/875	0/1754	0/522	
	c.1016dupA	0/971	0/969	0/1940	0/555	
	c.1961dupA	0/971	0/969	0/1940	0/555	
	c.2125_2126insA	0/965	0/965	0/1930	1/555	0.365
	c.2834_2836delGTAinsC	0/971	0/969	0/1940	0/555	
	c.3649_3650insA	0/971	0/963	0/1934	0/553	
	c.3756_3759delGTCT	0/971	0/969	0/1940	0/555	
	c.4327 C > T	2/971	0/969	2/1939	3/552	0.048
	c.5102_5103delTG	0/971	0/968	0/1939	0/555	
	c.1054 G > T	0/969	0/966	0/1935	0/553	
	c.5536 C > T	0/969	0/968	0/1937	0/555	
	All variants	2	0	2	4	0.017
*BRCA2*	c.2588dupA	0/971	0/968	0/1939	1/555	0.364
	c.2806_2809delAAAC	0/970	0/969	0/1939	0/555	
	c.3170_3174delAGAAA	1/945	0/952	1/1897	6/547	0.002
	c.3545_3546delTT	0/968	0/965	0/1933	2/550	0.132
	c.5857 G > T	1/970	0/966	1/1936	1/555	0.365
	c.6275_6276delTT	0/970	0/968	0/1938	0/555	
	c.8537_8538delAG	0/927	0/912	0/1839	7/536	<0.001
	c.9004 G > A	1/970	0/967	1/1937	0/554	
	All variants	3	0	3	17	<0.001
*PALB2*	c.2323 C > T	0/1000	0/1000	0/2000	0/582	
		0	0	0	0	

*The female controls and cases were compared using Fisher’s Exact Test.

The mean age of first invasive breast cancer diagnosis in *BRCA1/2* heterozygotes (43.6 years) was significantly younger than that of wild-types (53.0 years, *p* < 0.001). *BRCA1* and *BRCA2* heterozygotes had a mean age of diagnosis of 39.3 and 44.0, respectively (*p* = 0.344). When examining early-onset breast cancer cases, 12/21 (57.1%) of heterozygotes were diagnosed at 45 years or younger, compared with 113 of 534 (21.1%) in wild-types (*p* < 0.001). *BRCA1/2* heterozygotes were more than twice as likely to have a family history of cancer (12/21, 57.1%) than wild-types (134/534, 25.1%) (Table S1). 17 of 21 cases (81.0%) heterozygous for a variant had either a family history of any cancer or an early (<45 years) onset of disease. 12 of 113 (9.38%) of all early-onset cases carried a heterozygous variant in one of the 20 variants genotyped. Of the 28 cases who were diagnosed under 45 and had a reported family history of cancer, seven (25%) were heterozygous for a pathogenic founder variant.

Eleven of the *BRCA1/2* heterozygotes had other cancer diagnoses in their lifetime (Table S2). Seven had an asynchronous primary breast cancer: six contralateral and one ipsilateral. Two *BRCA2* heterozygotes had bilateral synchronous breast cancers. Three of the four cases also diagnosed with ovarian cancer in cases were *BRCA1/2* heterozygotes: one *BRCA1* and two *BRCA2* heterozygotes. Two heterozygotes were diagnosed with other cancers, in the uterus and adrenal gland. With the exception of the uterus cancer diagnosis, all secondary cancer diagnoses were subsequent to the heterozygote’s first invasive breast cancer.

## Discussion

Genotyping *BRCA1*, *BRCA2*, and *PALB2* French-Canadian breast cancer variants identified a pathogenic variant in 3.8% (21/555) of breast cancer cases unselected for age or family history and 0.2% (5/1940) of cancer-free controls. Our previous, smaller study of a series of breast cancer cases found 3.1% of French-Canadians cases had one of seven previously reported founder variants^15^, comparable to this study. Increasing the number of variants genotyped to twenty therefore did not increase sensitivity in this project.

Similar genotyping approaches have been taken in founder populations other than French Canadians. For instance, 7.1% of unselected Polish breast cancer cases carried one of five known founder variants in *BRCA1*^16^. In a study of 236 Ashkenazi Jewish women with breast cancer, one of three founder *BRCA1*/*BRCA2* variants was found in 25% of early-onset breast cancer cases (diagnosed under 45 years of age) and 18.5% of women diagnosed after 45 years of age^17^. Another study of 412 Ashkenazi Jewish women found 12% of breast cancers can be attributed to one of these three founder variants^18^. Further, 88 of 847 unselected Icelandic breast cancer cases (10.4%) carry a known founder variant^19^ (*BRCA2* NM 000059.3: c.771_775delTCAAA; Table S3). Unselected French Canadian cases therefore have a lower prevalence of *BRCA1* and *BRCA2* variants in comparison to other founder populations, likely due to multiple founder effects contributing to a founder population more heterogenous than others^20^. *BRCA1/2* frequencies in French Canadians appear, however, to be higher than in outbred populations: Genotyping of 1220 breast cancer cases unselected for family history, age, or ethnicity found 0.7% and 1.3% of *BRCA1* and *BRCA2* variant heterozygotes, respectively^21^.

This study found a 0.2% allele frequency for *BRCA1*, *BRCA2*, and *PALB2* founder variants in controls with no personal or family history of cancer. Notably, all five control individuals found to be heterozygotes were men (5/971, 0.5%). No cancer-free women were identified as heterozygotes (of 969), confirming the high penetrance of these variants. FLOSSIES is a large-scale sequencing project of 9884 European and African American women above age 70 with no personal history of cancer (www.whi.color.com). A search of likely pathogenic variants in FLOSSIES (frameshift, nonsense, and splice site variations) results in approximate allele frequencies of 0.03%, 0.13%, and 0.07% in *BRCA1*, *BRCA2*, and *PALB2*, respectively.

This study found no heterozygotes for *PALB2* c.2323 C > T. This was striking, as a previous study identified this variant in 0.6% (2/356) of French-Canadian early onset or familial breast cancer cases, though it was not identified in 6440 newborn controls from the Quebec City region^14^. The variant was identified twice more when screened for in 71 hereditary breast or breast/ovarian cancer families, indicating its association with breast cancer in French Canadians^22^. Another genotyping study found 0.7% of early-onset (diagnosed younger than 50 years of age) French Canadian breast cancer cases were heterozygous for this variant^23^. Our study was the first to genotype the variant in unselected breast cancer cases, with few early onset or familial cases, which likely accounts for the lower prevalence in this case series. *PALB2* is a rare, moderate to high penetrance gene^24^. The lifetime risk varies by family history^25^. Current guidelines suggest annual mammograms for patients with *PALB2* variants, with insufficient evidence to recommend risk-reducing mastectomy^26^. Moreover, as we did not observe this variant in any subjects in this study (reinforcing its rarity in Montreal French Canadians), screening for this variant in the French-Canadian population is not currently recommended.

Of all variants tested, only seven were identified in this case series. Notably, only *BRCA2* variants significantly associated with affected status. One study of early-onset breast cancer cases and newborn controls in Quebec found that majority of heterozygotes identified harboured a *BRCA2* founder variant rather than *BRCA1*^23^. Among newborns, similar variant frequencies were found in both *BRCA1* and *BRCA2*, comparable to control frequencies seen in this study. A similar pattern of only *BRCA2* variants was seen in 127 French Canadian breast cancer cases unselected for age or family history^15^. There is some evidence to suggest *BRCA2* variants may also be more prevalent in breast cancer cases outside of the French-Canadian population^18^. Taken together, it is presumed that the prevalence of pathogenic founder *BRCA2* variants is higher than that of *BRCA1* variants in French Canadians of Quebec.

Earlier age of cancer onset typically associates with genetic predisposition to breast cancer. Genotyping 20 previously reported variants in this French-Canadian series identified 12 among 125 breast cancer cases diagnosed younger than 45 years of age. Therefore, variant-specific testing was able to identify pathogenic variants in 9.6% of unselected breast cancer cases in Montreal French Canadians. This is consistent with our previous study, which found that testing for known founder variants in unselected French-Canadian cases was able to identify 13% of unselected breast cancer cases diagnosed between 25 and 40^23^. In another study genotyping only early-onset French Canadian cases, an identical percentage (13%; 8 of 61) diagnosed under 40 were found to have pathogenic variants in *BRCA1* or *BRCA2*^9^.

Our study identified heterozygotes for seven pathogenic variants that accounted for approximately 10% of early onset cases, and as high as 25% of early onset cases with a self-reported family history of cancer. The women with a founder variant in their germline likely were not tested previously and may not be aware of their elevated risk of breast cancer. With this knowledge, family members could be screened and heterozygous women would have the option to take preventive measures for breast cancer. Prophylactic mastectomies are likely effective in reducing breast cancer incidence^27^, but this is dependent on patients opting to undergo the surgery. In a recent study looking at 128 breast cancer patients in Korea with a *BRCA1* or *BRCA2* carriers, only 8 chose to undergo contralateral prophylactic mastectomies^28^.Among French-Canadians of Quebec who carry *BRCA1* or *BRCA2* variants, preventive options including bilateral prophylactic mastectomies are less popular than in other regions of Canada^29^, with only 8% of Quebec women heterozygous for a pathogenic variant opting for the surgery compared to 22–46% in other regions of Canada. Despite this, population-wide genetic screening could identify up to 10% of early-onset cases prior to a diagnosis. This would provide more options for these women as well as potentially increasing awareness of these options in the general population and improving the acceptability of such preventive intervention measures among Quebec women.

Population-based screening for cancer predisposition has previously been tested in founder populations such as Ashkenazi Jews and Poles. Testing for two to three founder variants in each population served as a cost-effective method in each case to screen large cohorts of individuals and was able to identify up to 4.5% of individuals as *BRCA1* or *BRCA2* heterozygotes. Notably, many of these heterozygotes would not have been identified following government-suggested guidelines for genetic testing (such as the NCCN in the United States). Large-scale genotyping of founder variants in the French-Canadian population may therefore be a feasible choice that would be more effective in identifying heterozygotes than the current standard of evaluating family history alone^30^.

Health economic analyses of population-wide genetic screening found screening for founder variants is likely affordable for some healthcare systems^30^. Preventive strategies such as prophylactic mastectomies and salpingo-oophorectomies of known heterozygotes are also more cost-effective than typical MRI-based screening. Psychological impact is also a factor to be considered: Are patients relieved or worried when receiving a positive test result for pathogenic *BRCA1/2* variants? One study found that the psychological burden associated with population-based screening (i.e. depression, anxiety, distress) is not significantly different from that of standard family-based screening^31^. Another study found that Black women with breast cancer will only choose to have *BRCA1/2* testing if the test is both necessary and affordable^32^. More work is needed to examine the accessibility and affordability of population-based screening.

In our study, one in 10 early-onset cases was heterozygous for one of seven founder variants. Providing population-wide screening to French Canadians would be feasible, and these numbers suggest the impact on breast cancer incidence would be positive, though economic costs and psychological outcomes would need to be evaluated.

## Methods

### Study subjects

DNA from peripheral blood of 588 breast cancer cases and 2,000 cancer-free controls were studied (Table 2). Breast cancer cases were ascertained between years 2000 and 2016 from the Centre Hospitalier de l’Université de Montréal (CHUM), Montreal, Quebec, Canada via a Cancer Research Network (RRCancer, http://rrcancer.ca/en/home/) solid tumor breast cancer biobank. Eligible breast cancer cases included women with a surgically-resectable invasive breast cancer diagnosis at under 70 years of age and French-Canadian descent (as determined by a review of family names; Table 3). Information regarding prior and subsequent cancer diagnoses, hormone receptor status, axillary lymph node status, grades, tumour size, and morphologies was collected for most breast cancer diagnoses, where possible. Family history of cancer in any relatives was self-reported.

**Table 3:** Filtering process for genotyped samples.

Filter	Cases	Controls
Initial samples from biobanks	588	2,000
Removed for exceeding age limits	2	0
Removed for duplication of samples	1	0
Removed because of low DNA quality for genotyping	30	60
Final number of samples genotyped	555	1,940

Cancer-free controls included 1,000 women and 1,000 men aged 40 to 70 that were ascertained between 2009 and 2015 through the CARTaGENE biobank^33^ (https://www.cartagene.qc.ca/en/home). Controls were French Canadian as determined by French first language, Canadian origin of all four grandparents and a city of birth within Quebec. Participants reported no personal history of cancer and no cancer in any first-degree relatives. Controls for this study were restricted to individuals recruited within the greater Montreal area (Table 4).

**Table 4:** Summary of cases and controls series for French-Canadian *BRCA1/2/PALB2* genotyping.

Characteristic	Cases	Controls
Dates of ascertainment	2000–2016	2009–2015
Ages at ascertainment (mean)	32–89 (60.7)	40–70 (53.2)
Ages of first invasive breast diagnosis (mean)	25–69 (52.7)^*^	–
Location of ascertainment	Montreal, QC	Montreal, QC
Ethnicity	French-Canadian^†^	French-Canadian‡
Personal history of cancer	At least one invasive breast cancer	None^§^
Family history of cancer	55% no, 27% yes, 19% unknown^§^	None^§^

*Intervals between ages at first invasive breast cancer and ascertainment range from 0 to 11 years (Mean = 0.67, S.D. = 5.36).

^†^As determined by a review of last names.

^‡^As determined by French first language, Canadian origin of all four grandparents, and a city of birth located in Quebec.

^§^Self-reported data.

All case and control samples were collected through informed consent approved by institutional review boards (McGill University Institutional Review Board, study A08-M61-098). Methods were carried out in accordance with relevant guidelines and regulations.

### *BRCA1/BRCA2/PALB2* genotyping

Twenty previously reported^7–14^ SNP/indel pathogenic variants were genotyped in breast cancer cases and cancer-free controls. These included 11 *BRCA1* variants, 8 *BRCA2* variants, and 1 *PALB2* variant. DNA samples were subjected to whole genome amplification using the Qiagen REPLI-g Mini Kit according to manufacturer’s instructions. Genotyping was performed using the Sequenom iPLEX Gold Genotyping Technology^34^ with at least 600 ng of whole-genome-amplified DNA per sample through the McGill University and Genome Quebec Innovation Centre. Exactly 346 samples failed the Sequenom assay for *PALB2* c.2323 C > T (p.Gln775Ter) only. To resolve this deficiency, genotyping was complemented by a *PALB2* exon 5 high resolution melting assay for the variant, as previously described^35^. Genotyping failed for 90 samples at all loci (30 cases, 60 controls); these samples were removed from the analysis.

### Validation of heterozygotes

Select germline heterozygotes were confirmed by amplifying the region of interest by polymerase chain reaction (PCR), followed by Sanger sequencing. PCR reactions were performed in 25 uL volumes containing 20 ng genomic DNA; 10 x PCR buffer with 15 mM magnesium chloride (Qiagen); 100 uM of each dATP, dCTP, dGTP, and dTTP; 25–35 nmoles of each primer used in Sequenom genotyping; and 2.5 units/uL HotStarTaq DNA polymerase (Qiagen). Reactions were amplified in a Bio Rad DNAEngine^®^ Peltier Thermal Cycler for 35 cycles at 95 °C for 30 seconds, 60 °C for 30 seconds, and 72 °C for 30 seconds, followed by 72 °C for 3 minutes. PCR products were diluted 2X with formamide and bromophenol blue loading buffer, then loaded on a 1.5% agarose gel with RedSafe Nucleic Acid Staining Solution. PCR products were then Sanger sequenced through the McGill University and Genome Quebec Innovation Centre with the forward primers used in Sequenom genotyping.

### Statistical analysis

All comparisons related to breast cancer cases are based on the first invasive breast cancer diagnosis for each patient. Comparisons of proportions were conducted using Fisher’s Exact Test, using *p* < 0.05 as a significance cut-off. Comparisons of means were conducted using a two-tailed T-test, using p < 0.05 as a significance cut-off. All statistical analyses were conducted in RStudio Version 1.1383.

### Financial support

This study was supported by a grant from the Quebec Breast Cancer Foundation (QBCF) to WDF. SB received personal financial support from a Canadian Institute for Health Research CGS-M grant and a Research Institute of the McGill University Health Centre Masters Studentship Award.

## Supplementary information

Supplementary information.
