# Supplementary material for: Founder BRCA1/BRCA2/PALB2 pathogenic variants in French-Canadian breast cancer cases and controls
Source: Sci Rep. 2020 Apr 16;10:6491. doi: 10.1038/s41598-020-63100-w (PMC7162921; doi:10.1038/s41598-020-63100-w)
Supplement: Supplementary file 1 — Supplementary information. [file 41598_2020_63100_MOESM1_ESM.docx]

**Supplementary Material**

**Table S1.** Early onset and family history information for heterozygote and wild-type cases.

| Characteristic | Heterozygous Cases (n = 21) | | | Wild-Type Cases  (n = 534) | *p*-value^*^ |
| --- | --- | --- | --- | --- | --- |
|  | All | BRCA1  (n = 4) | BRCA2 (n = 17) |  |  |
| Early Onset (Diagnosed < 45 years) | 12 | 2 | 10 | 113 | 0.001 |
|  |  |  |  |  |  |
| Family History |  |  |  |  |  |
| Yes | 12 | 2 | 10 | 134 | 0.004 |
| No | 5 | 1 | 4 | 298 | 0.006 |
| N/A | 4 | 1 | 3 | 102 |  |
|  |  |  |  |  |  |
| Early Onset and Family History | 7 | 1 | 6 | 21 | 0.001 |

*p*-values were calculated using Fisher’s Exact Test, comparing wild-type to *BRCA1* and *BRCA2* heterozygous cases combined

**Table S2.** Synchronous and asynchronous diagnoses of breast cancer cases heterozygous for *BRCA1/2* variants.

| Gene | Mutation | Age of Diagnosis | | Diagnosis | Laterality | | |
| --- | --- | --- | --- | --- | --- | --- | --- |
| *BRCA1* | 4446C>T | 33 | Breast | | | Left |  |
|  |  | 60 | Breast | | | Right |  |
|  |  | 35 | Breast | | | Left |  |
|  |  | 44 | Breast | | | Right |  |
|  |  | 44 | Breast | | | Left |  |
|  |  | 38 | Breast | | | Left |  |
|  |  | 43 | Breast | | | Right |  |
|  | 2244insA | 51 | Breast | | | Right |  |
|  |  | 52 | Ovary | | | Left |  |
|  |  | 57 | Breast | | | Left |  |
| *BRCA2* | 8765delAG | 43 | Breast | | | Left |  |
|  |  | 53 | Breast | | | Left |  |
|  |  | 39 | Breast | | | Right |  |
|  |  | 39 | Breast | | | Left |  |
|  |  | 46 | Ovary | | | N/A |  |
|  |  | 48 | Breast | | | Left |  |
|  |  | 48 | Breast | | | Right |  |
|  | 3398del5 | 33 | Breast | | | Right |  |
|  |  | 40 | Ovary | | | Left |  |
|  |  | 51 | Breast | | | Left |  |
|  | 3773delTT | 53 | Uterus | | | N/A |  |
|  |  | 56 | Breast | | | Left |  |
|  |  | 57 | Adrenal Gland | | | Left |  |
|  |  | 57 | Adrenal Gland | | | N/A |  |
|  |  | 54 | Breast | | | Right |  |
|  |  | 59 | Breast | | | Left |  |
|  | 2816insA | 51 | Breast | | | Right |  |
|  |  | 63 | Breast | | | Left |  |
|  |  | 68 | Breast | | | Left (recurrence) |  |

**Table S3.** Genotyping of unselected breast cancer cases in founder populations.

| Population | Number of breast cancer cases screened | Number of founder variants genotyped | Proportion of founder heterozygotes (%) | Reference |
| --- | --- | --- | --- | --- |
| French Canadians |  |  |  |  |
| This study | 555 | 20 | 3.8 | –––– |
| Chappuis et al | 127 | 7 | 3.1 | Chappuis et al (2001) |
| Other Founder Populations |  |  |  |  |
| Polish | 1164 | 12 | 7.1 | Szwiec et al (2015) |
| Ashkenazi Jewish | 412 | 3 | 12 | Warner et al (1999) |
| Iceland | 847 | 1 | 10.4 | Tryggvadottir et al (2006) |

**Table S4.** Clinical features of breast cancers for heterozygous and wild-type cases.

| Characteristic | Heterozygote Cases (n = 21) | | | Wild-Type Cases (n =534) | *p*-value^*^ |
| --- | --- | --- | --- | --- | --- |
|  | All | BRCA1 (n = 4) | BRCA2 (n = 17) |  |  |
| Breast Diagnoses |  |  |  |  |  |
| Ductal Carcinoma (IDC) | 19 | 4 | 15 | 403 | 0.388 |
| Lobular Carcinoma (ILC) | 2 | 0 | 2 | 53 |  |
| In Situ | 0 | 0 | 0 | 23 |  |
| Unknown |  |  |  | 55 |  |
| Axillary Lymph Node Status |  |  |  |  |  |
| Positive | 5 | 0 | 5 | 177 | 0.511 |
| Negative | 12 | 4 | 8 | 302 |  |
| Unknown | 4 | 0 | 4 | 55 |  |
| Grade |  |  |  |  |  |
| 3 | 10 | 1 | 9 | 121 | 0.018 |
| 2 | 3 | 0 | 3 | 213 |  |
| 1 | 2 | 1 | 1 | 86 |  |
| Unknown | 6 | 2 | 4 | 114 |  |
| T Stage |  |  |  |  |  |
| T1 | 8 | 2 | 6 | 271 | 0.372 |
| T2 | 5 | 0 | 5 | 172 |  |
| T3 | 1 | 0 | 1 | 22 |  |
| T4 | 0 | 0 | 0 | 5 |  |
| Unknown | 3 | 1 | 2 | 64 |  |
| Estrogen Receptor |  |  |  |  |  |
| Positive | 10 | 0 | 10 | 410 | 0.001 |
| Negative | 7 | 2 | 5 | 72 |  |
| Unknown | 4 | 0 | 4 | 52 |  |
| Progesterone Receptor |  |  |  |  |  |
| Positive | 8 | 0 | 8 | 380 | 0.001 |
| Negative | 9 | 2 | 7 | 102 |  |
| Unknown | 4 | 2 | 2 | 52 |  |
| HER2 Overexpression |  |  |  |  |  |
| Positive | 0 | 0 | 0 | 70 | 0.251 |
| Negative | 15 | 2 | 13 | 382 |  |
| Unknown | 5 | 2 | 3 | 82 |  |
| Triple Negative Cases | 6 | 2 | 4 | 39 | 0.004 |

^*^ *p*-values were calculated using Fisher’s Exact Test, comparing wild-type, cases heterozygous for *BRCA1* variants, and cases heterozygous for *BRCA2* variants

**Table S5**. Primer sequences used in PCR and Sequenom genotyping of breast cancer cases and cancer-free controls.

| Gene | HGVS Nomenclature | Forward Primer | Reverse Primer |
| --- | --- | --- | --- |
| *BRCA1* | c.962G>A | ACCTTTTTTTCTGTGCTGGG | AAGCAAACAGCCTGGCTTAG |
|  | c.1016dupA | AATGATAGGCGGACTCCCAG | AATGATAGGCGGACTCCCAG |
|  | c.1961dupA | AAGCCCACCTAATTGTACTG | TTGTAGGTTTCTGCTGTGCC |
|  | c.2125_2126insA | CTTGGAAGGCTAGGATTGAC | ATACTTTCCCAGAGCTGAAG |
|  | c.2834_2836delGTAinsC | TCCTGTGGTTGGTCAGAAAG | GTTTCGTTGCCTCTGAACTG |
|  | c.3649_3650insA | AAGCAGGGAAGCTCTTCATC | CACCCATACACATTTGGCTC |
|  | c.3756_3759delGTCT | TACTAGGCATAGCACCGTTG | ATTACCTGGTTACTGCAGTC |
|  | c.4327C>T | TTTGGCCAACAATACACACC | TTTGGCCAACAATACACACC |
|  | c.5102_5103delTG | GCTCTTTAGCTTCTTAGGAC | CATTTTCCTCCCGCAATTCC |
|  | c.1054G>T | AATGATAGGCGGACTCCCAG | CTAGGATTCTCTGAGCATGG |
|  | c.5536C>T | AGCAATTGGGCAGATGTGTG | ATCAGGTAGGTGTCCAGCTC |
| *BRCA2* | c.2588dupA | AGAGTAGCATCACCTTCAAG | GTTCTTCAGAGTCTGGATTG |
|  | c.2806_2809delAAAC | TGACTTGTGTAAACGAACCC | CCTCTGCAAGAACATAAACC |
|  | c.3170_3174delAGAAA | TCCTACTAGTTTAGCTTGTG | TCCTACTAGTTTAGCTTGTG |
|  | c.3545_3546delTT | CATGTCATAATGAATGCCCC | TCAACAGGCCAGCAAACTTC |
|  | c.5857G > T | TATGTCTGGATTGGAGAAAG | ACTGACTTATGAAGCTTCCC |
|  | c.6275_6276delTT | GGGAGTGTTAGAGGAATTTG | CTCTTATCAACACGAGGAAG |
|  | c.8537_8538delAG | AGTCTCTTTTGTTGGGCCTC | GATGGAGAAGACATCATCTG |
|  | c.9004G > A | TTGTCGCTGCTAACTGTATG | CTGAGTATTTGGCGTCCATC |
| *PALB2* | c.2323C>T | AAGACTCAGTCTGTCTTGCC | TATGTGGTTTTGCTGGGCTG |
